# A semi-automated virtual workflow solution for the design and production of intraoral molding plates using additive manufacturing: the first clinical results of a pilot-study

**DOI:** 10.1038/s41598-018-29959-6

**Published:** 2018-08-07

**Authors:** Florian D. Grill, Lucas M. Ritschl, Franz X. Bauer, Andrea Rau, Dominik Gau, Maximilian Roth, Markus Eblenkamp, Klaus-Dietrich Wolff, Denys J. Loeffelbein

**Affiliations:** 1 Department of Oral and Maxillofacial Surgery, Technische Universität München, München, Germany; 2 Institute of Medical and Polymer Engineering, Technische Universität München, München, Germany; 3 Department of Oral and Maxillofacial Surgery, Friedrich Alexander Universität Erlangen-Nürnberg, Erlangen, Germany; 4 Department of Oral and Maxillofacial Surgery, Helios Hospital Munich West, Teaching Hospital of Ludwig-Maximilians-Universiität München, München, Germany

**Keywords:** Health care, Paediatric research

## Abstract

Computer-aided design and computer-aided manufacturing (CAD/CAM) technology has been implemented in the treatment of cleft lip and palates (CLP) by several research groups. This pilot study presents a technique that combines intraoral molding with a semi-automated plate generation and 3D-printing. The clinical results of two intraoral molding approaches are compared. This is the first clinical investigation of semi-automated intraoral molding. Our study included newborns with unilateral CLP. Plaster models were digitalized and measured by two independent observers. Two methods of CAD/CAM-assisted intraoral molding were compared: (i) stepwise manual design of molding plates (conventional CAD/CAM-intraoral molding) and (ii) a semi-automated approach with an automated detection of alveolar ridges (called RapidNAM) assisted by a graphical user interface (GUI). Both approaches significantly narrowed the clefts and resulted in a harmonic alveolar crest alignment. The GUI was easy to use and generated intraoral molding devices within minutes. The presented design solution is an efficient technical refinement with good clinical results. The semi-automated plate generation with a feasible GUI is fast but allows individual adaptations. This promising technique might facilitate and foster the more widespread use of CAD/CAM-technology in intraoral molding therapy.

## Introduction

Nasoalveolar molding (NAM) is a presurgical orthofacial treatment modality for newborns with cleft lip and palates (CLP). It starts with the insertion of the first drinking plate, which is adjusted according to the alveolar development and extended with a nasal stent during the course of treatment. The early phase of treatment seems determining^1^. NAM is completed when primary closure of the lip is performed surgically^2^. Nevertheless, NAM is criticized as being a time-consuming treatment modality involving weekly adjustments and several impression-takings when new plates are needed (burden of care). Furthermore, it is only offered in specialized centers^3,4^. However, NAM is associated with promising long-term trends leading to reduced secondary corrections and a consecutive reduction of costs^5–8^. Therefore, the introduction of computer-aided design and computer-aided manufacturing (CAD/CAM) technology has been adopted in NAM treatment not only to facilitate the production modality itself, but also to reduce impression-takings. It can potentially increase its accessibility by providing an easy-to-use software solution and the possibility to outsource the production procedure. The challenge remains to create an efficient and feasible virtual workflow, since simple, low-cost, and accessible software solutions are still lacking. The means of rapid prototyping allows efficient production but only once the plate has been created digitally^9^. Despite this, rapid prototyping still seems promising with regard to efficiency giving comparable clinical results to traditional methods. Moreover, plate generation differs from center to center and is based on the experience of each research group^10,11^. However, even the conventional molding technique has different variations with respect to the closure of the alveolar cleft^12–16^. The digital environment requires knowledge of the usage of 3D-programs and can be the source of pitfalls for an inexperienced user^11^. Using CAD/CAM-technology the costs for a molding therapy could be lowered significantly since the presented intraoral molding device already provides the technical requirements for a consecutive nasal molding. The purpose of the presented study has been to analyze the effectiveness of the new semi-automated intraoral molding plate generation (called RapidNAM) and to describe the virtual workflow.

## Patients, Materials and Methods

### Informed consent

All interactions with each patient in this study were performed with parental informed consent.

### Treatment methods

Healthy newborns with unilateral CLP (n = 14) were included in the study. Two groups were formed: one group was treated with conventional CAD/CAM-intraoral molding plates as published previously with digitally designed intraoral molding plates serving as a reference group^9,17^ and the other group with RapidNAM-plates. In both groups, impressions were taken from the upper jaw within the first few days of life and at the end of molding therapy when primary lip closure was performed at the age of approximately 3–4 months. The casts were digitalized with a 3D triangulation scanner (3Shape D500, 3Shape; Copenhagen, Denmark)^9,11,18^.

As commonly done at our treatment center using conventional molding devices in this study, both treatment groups attended weekly clinical controls as well. If necessary, plates were adjusted. Buccal taping and the taping of the upper lip across the cleft were performed in the conventional way in both groups by using the Grayson-technique^15^.

### Conventional CAD/CAM-molding

In the CAD/CAM-molding group, the alveolar ridge was given space for expansion by the stepwise molding of each plate virtually upon a digitalized plaster model creating a series of plates based on only one cast model. In order to grant the alveolar ridge enough space for expansion, an empirical growth factor was implemented. Moreover, the retention pin had to be inserted by the user by a Boolean operation (Geomagic^®^ Studio 12, Morrisville, NC, USA)^11^.

### RapidNAM and semi-automated plate generation

**Figure 1:**
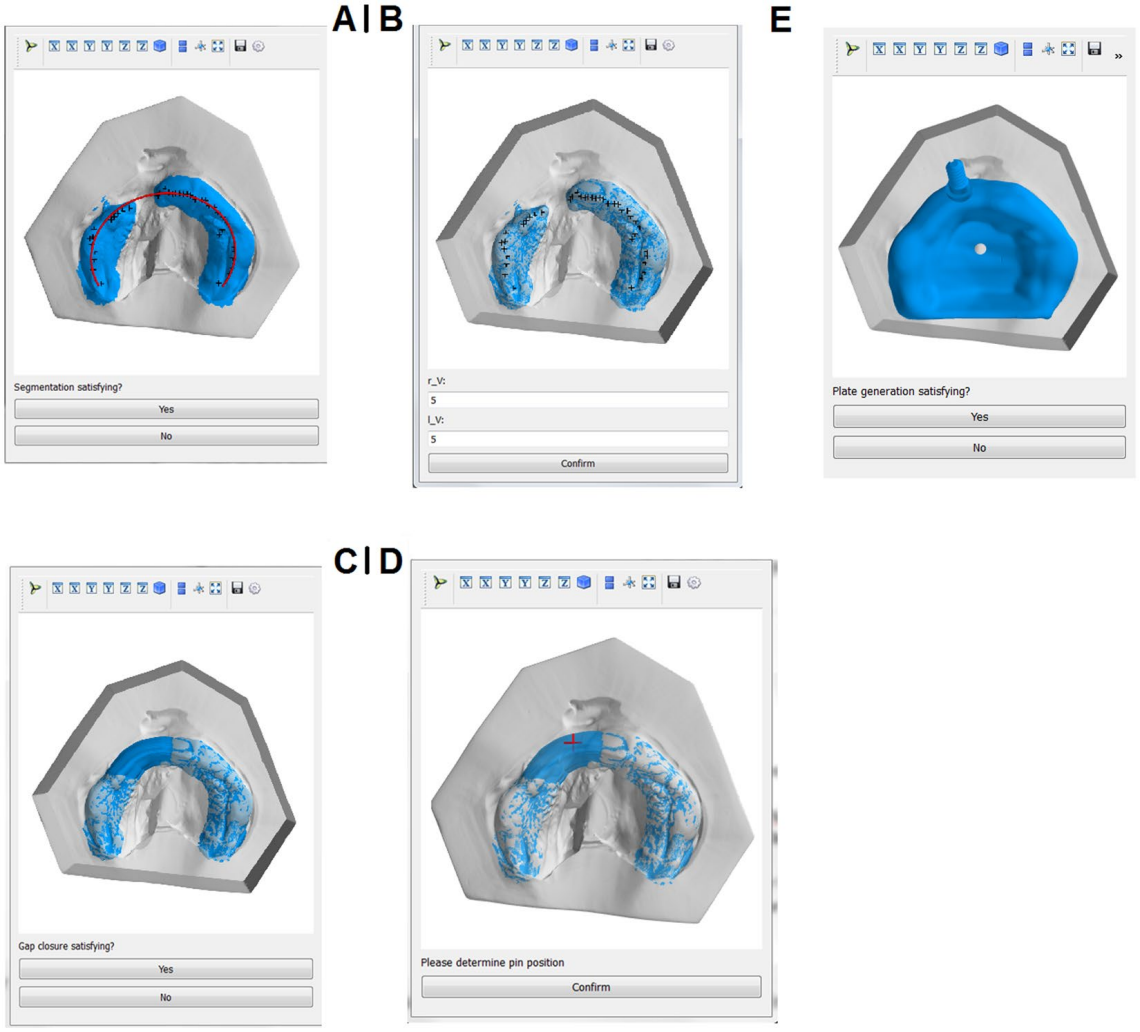
Graphical User Interface for the design of RapidNAM devices. (**A**) Automated detection of alveolar crest. (**B**) Selection of bridging area. (**C**) Gap closure. (**D**) Pin positioning. (**E**) Virtual plate.

The second group was treated with RapidNAM, a semi-automated technique of intraoral molding, which is also based on only one impression-taking to create a series of molding plates. The monthly growth rate was determined in an anatomical study of 32 healthy newborns. The algorithm automatically detected the alveolar ridge^19,20^. For this reason, a graphical user interface (GUI) was created (Fig. 1A–E). Our employed GUI was designed for the treating physicians, so that individual soft tissue situations could be considered in the design of the RapidNAM-plates on site. The following steps were necessary to create a series of molding plates, although the number of plates can be varied as clinically needed: (i) digitalization of the plaster models, (ii) ellipse-finding of the alveolar ridge by the algorithm with confirmation and correction if needed (Fig. 1A), (iii) selection of the areas on the greater and smaller segments, if gap closure has to be performed virtually with confirmation or correction of needed (Fig. 1B,C), (iv) positioning of the pin^21^ and automated integration of a ventilation whole (Fig. 1D), (v) smoothing of the plate (1E) surface, if necessary^20^.

### Measuring methods

**Figure 2:**
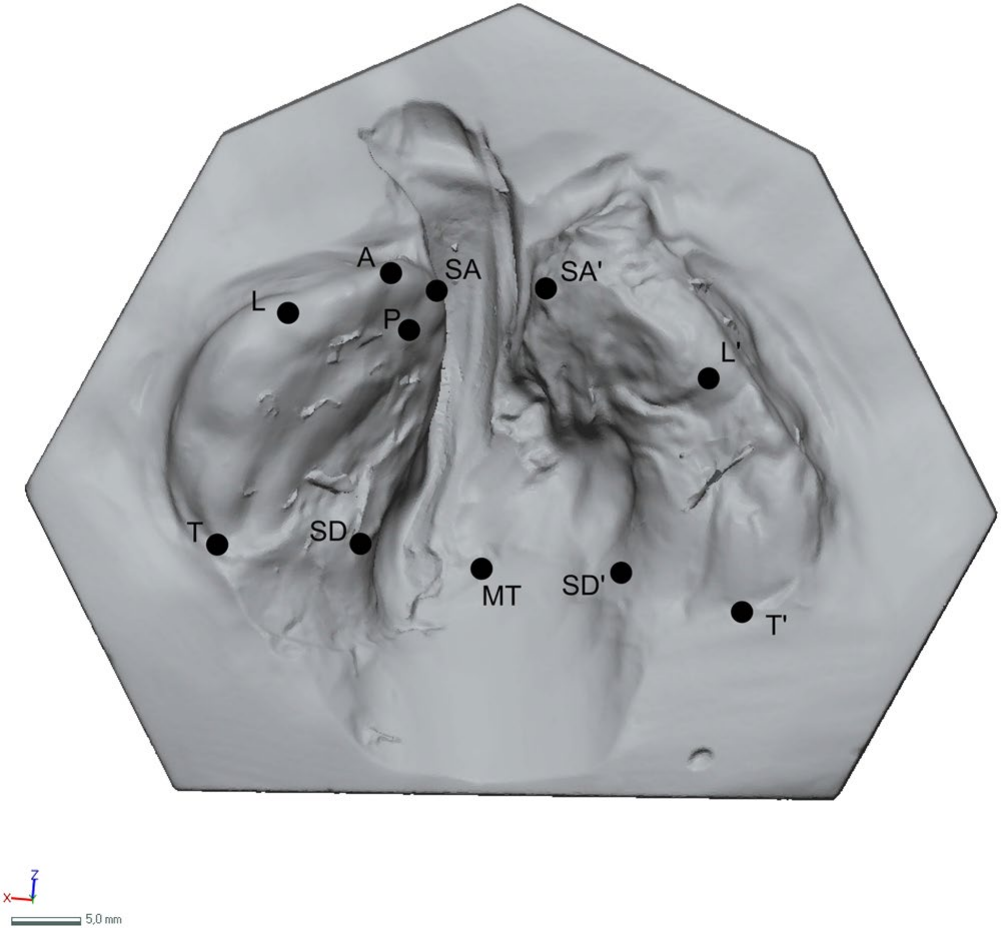
Selected landmarks.

The digitalized plaster models pre- and post-molding therapy of both groups were virtually analyzed by using selected landmarks as previously described^19,22^. For this purpose, the plaster models of CAD/CAM-molding-treated children were re-measured. Two independent observers selected the following landmarks: the most anterior point on the greater segment (A), the center of the papilla incisiva (P), the points that have the shortest connection line passing over the anterior cleft (SA and SA’), the sulcus lateralis points (L and L’), the tuberal areas (T and T’), the dissecting point on connecting line between tuberal areas (MT), the points that have the shortest distance passing over the cleft posterior on the hard palate (SD and SD’) (Fig. 2).

### Statistical analysis

Statistical analysis was carried out by using the R statistical environment with the user interface R Studio. For calculating differences between the initial and final impression-taking, the Wilcoxon-signed rank test and the Wilcoxon exact test were used. A p-value of < 0.05 was considered statistically significant. The plots show box plots with the upper and lower borders indicating the 1^st^ and 3^rd^ quartiles. The median is represented by the bar within the box^23,24^.

### Data availability

Data used for analysis in this study will be made available after publication.

## Results

In this pilot study, 14 newborns with CLP were included and treated with CAD/CAM-intraoral molding plates (n = 7) or RapidNAM-plates (n = 7). One patient dropped out of the group using RapidNAM-plates completely because of difficulties in parental taping application. In this case, a regular drinking-plate was used. One second impression taken on the operation day for primary lip closure was excluded because of artifacts at the landmark positions.

### RapidNAM Graphical User Interface

The former manual plate molding method, which was based on an empiric growth factor, involved several steps of plate expansions involving the use of special 3D software. Next to the manual insertion of the retention pin, this method was time-consuming with a duration of up to 1.5 hours per plate series^9,11^. The RapidNAM-software solution for a virtual semi-automated intraoral molding plate generation created a virtual series of plates within 10–15 minutes versus approximately one and a half hours for a virtual series of plates using the conventional and manual CAD/CAM-method. The areas on both alveolar segments at which the bridging is supposed to take place have to be selected manually. However, this is nevertheless intended to be non-automated in order to consider individual differences in size and configuration of the segments. Since the program is still a preliminary solution, the stl.-files have to be copied in designated folders manually. In one case, the stl.-file was erroneous and did not fit the alveolar ridge. After trouble-shooting, we found irregularities in the first impression-taking and could produce a well-fitting series of plates after re-taking the impression.

### Alveolar ridge development

#### Conventional CAD/CAM-intraoral molding

**Figure 3:**
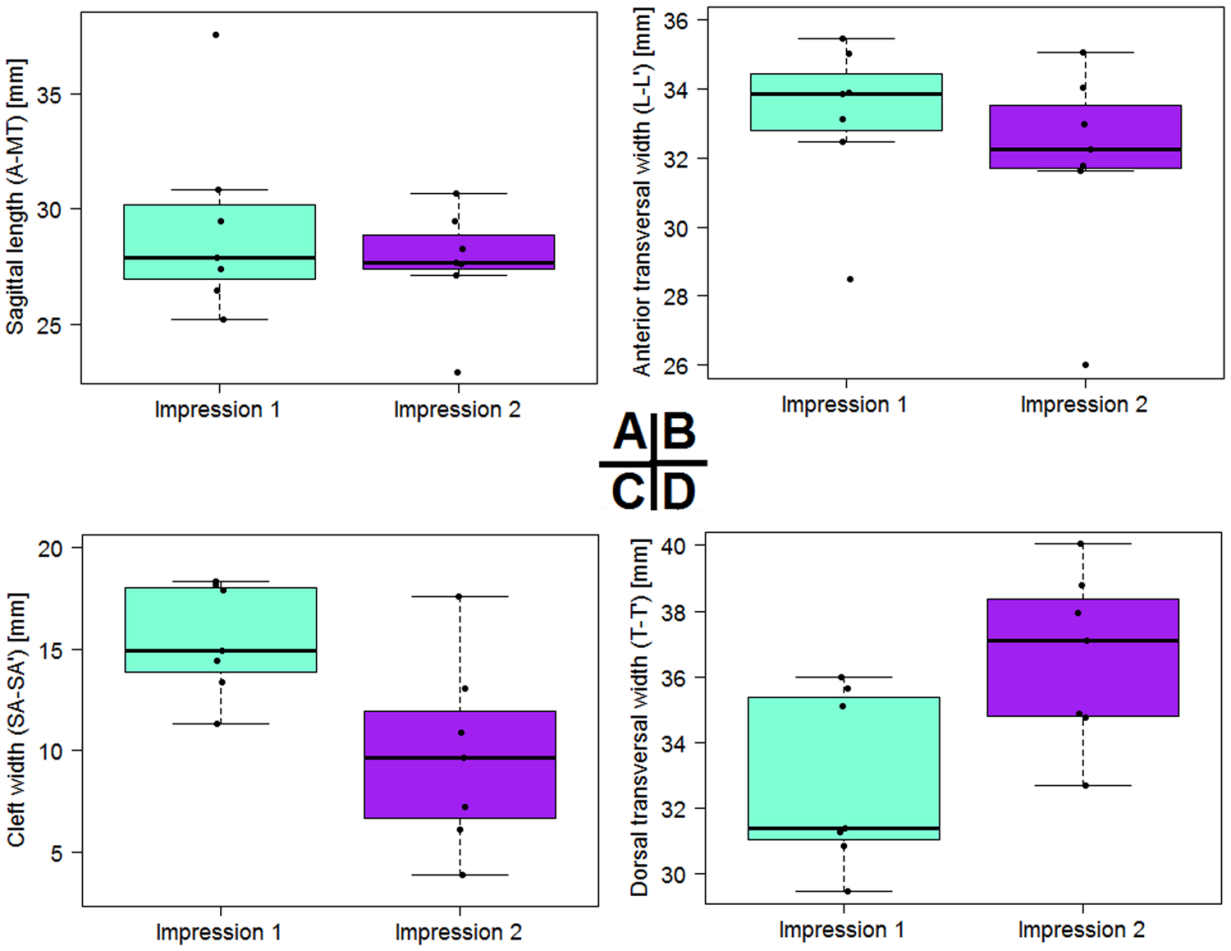
Statistical results before and after conventional CAD/CAM-molding treatment. (**A**) Longitudinal dimensions. (**B**) Transversal dimensions between sulcus lateralis points. (**C**) Cleft reduction. (**D**) Transversal dimension between the tubera.

The entire length, measured from point A to MT, remained nearly unchanged and reached a mean of 29.3 mm before lip closure (Fig. 3, Table 1). The transversal dimensions, measured as the distance from L to L’, experienced an approximation of 1.2 mm, starting with a mean of 33.2 mm and ending up with 32.0 mm. The cleft width, described by the distance between SA and SA’, had a mean of 15.5 mm and experienced a significant reduction of 5.7 mm (p = 0.011, Table 2). The posterior dimensions, measured on the basis of the points T and T’, increased starting with a mean of 32.8 mm and expanding to 36.6 mm in the observation period. The posterior cleft dimension remained nearly unchanged with a slight reduction of 0.7 mm. All measured distances and difference are shown in Table 1.

**Table 1:** Distances between reference points with differences before and after conventional CAD/CAM-molding therapy.

DISTANCE	MIN.	1ST QUARTILE	MEDIAN	MEAN	3RD QUARTILE	MAX.	SD
A - MT IMPRESSION 1	25.2	26.9	27.9	29.3	30.2	37.6	4.1
A - MT IMPRESSION 2	22.9	27.4	27.6	27.7	28.9	30.7	2.5
A - MT DIFFERENCE	−14.7	−2.2	0.2	−1.6	1.7	4.2	6.3
L - L’ IMPRESSION 1	28.5	32.8	33.9	33.2	34.5	35.5	2.3
L - L’ IMPRESSION 2	26.0	31.7	32.2	32.0	33.5	35.1	2.9
L - L’ DIFFERENCE	−7.1	−2.7	−1.0	−1.2	−0.9	6.6	4.1
SA - SA’ IMPRESSION 1	11.3	13.9	14.9	15.5	18.0	18.3	2.7
SA - SA’ IMPRESSION 2	3.9	6.7	9.6	9.8	12.0	17.6	4.6
SA - SA’ DIFFERENCE	−11.1	−7.2	−7.0	−5.7	−3.4	−0.7	3.6
T - T’ IMPRESSION 1	29.4	31.1	31.4	32.8	35.4	36.0	2.7
T - T’ IMPRESSION 2	32.7	34.8	37.1	36.6	38.4	40.1	2.6
T - T’ DIFFERENCE	1.5	3.4	3.7	3.8	4.0	6.6	1.5
SD - SD’ IMPRESSION 1	16.8	18.2	20.6	21.2	24.2	25.9	3.7
SD - SD’ IMPRESSION 2	16.1	16.5	20.0	20.5	22.7	28.9	4.7
SD - SD’ DIFFERENCE	−4.4	−3.0	−0.6	−0.7	1.2	3.9	3.1

**Table 2:** P-values for differences in distances before and after conventional CAD/CAM-molding and RapidNAM-molding therapy.

CAD/CAM NAM		RapidNAM	
DISTANCES	P-VALUES	DISTANCES	P-VALUES
A - MT	0.589	A - MT	0.710
SA - SA’	0.041	SA - SA’	0.011
SD - SD’	0.699	SD - SD’	0.620
T - T’	0.132	T - T’	0.053
L - L’	0.132	L - L’	0.318
A - P	0.937	A - P	0.805
SA - SD	1.000	SA - SD	0.209

#### RapidNAM-intraoral molding

**Figure 4:**
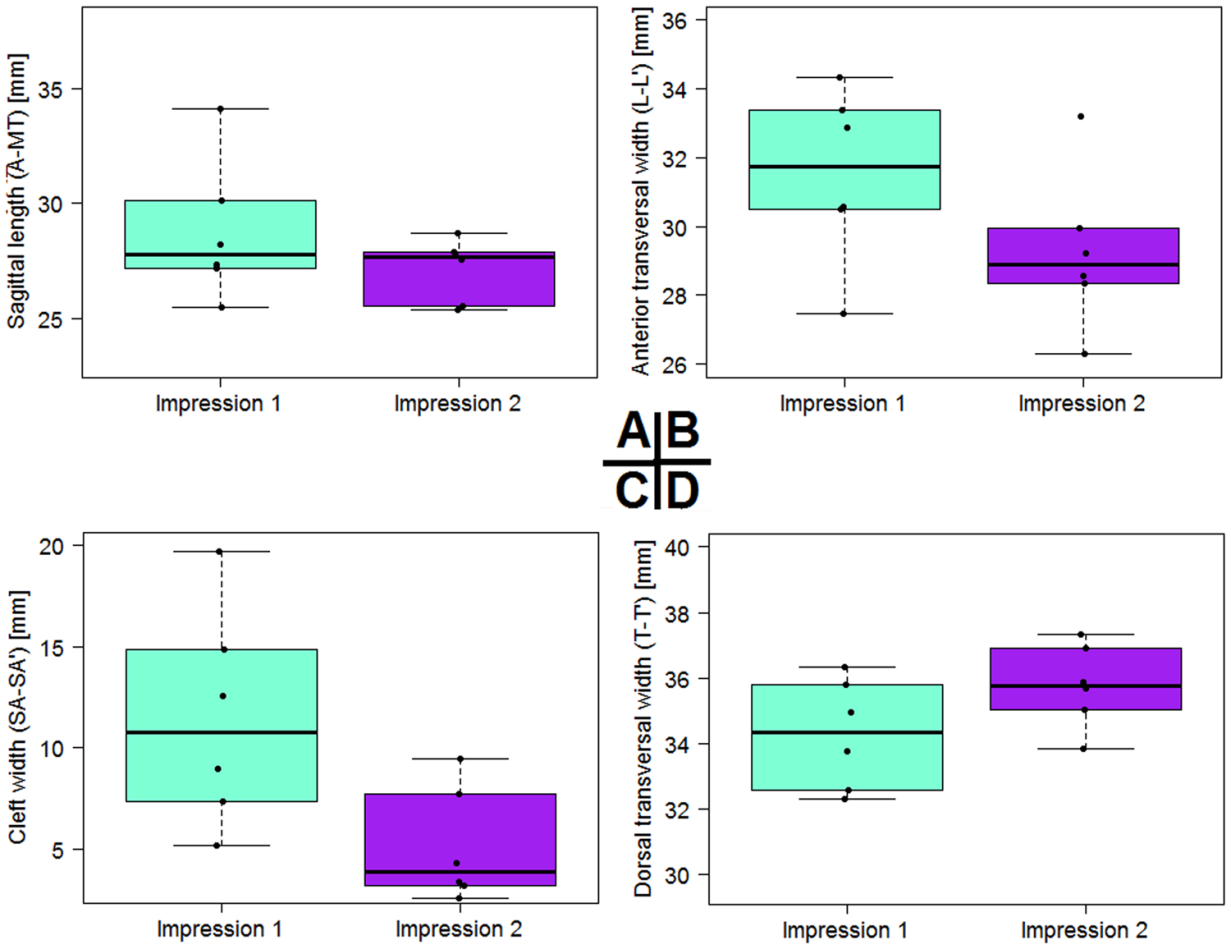
Statistical results before and after RapidNAM-molding treatment. (**A**) Longitudinal dimensions. (**B**) Transversal dimensions between sulcus lateralis points. (**C**) Cleft reduction. (**D**) Transversal dimension between the tubera.

The overall longitudinal length initially had means of 28.7 mm and 27.1 mm after NAM (Fig. 4). The difference was ca. 1.6 mm. The transversal dimensions decreased from an L-L’ mean distance of 31.5 mm to 29.2 mm. The distance between both alveolar segments represented by points SA and SA’ was significantly narrowed after RapidNAM-therapy. The cleft closure was statistically significant (p = 0.041, Table 2). The initial mean cleft widths had a mean of 11.4 mm, but the width was 5.1 mm when primary lip closure was performed surgically. This is a mean reduction of 6.4 mm. Transversal dimensions at the tuberal level experienced a slight expansion with a mean of 1.5 mm. The posterior cleft distances were also narrowed from a median 21.1 mm to 20.7 mm. All measured distances and differences are shown in Table 3.

**Table 3:** Distances between reference points with differences before and after RapidNAM-molding therapy.

DISTANCE	MIN.	1ST QUARTILE	MEDIAN	MEAN	3RD QUARTILE	MAX.	SD
A - MT IMPRESSION 1	25.5	27.2	27.8	28.7	29.7	34.1	3.0
A - MT IMPRESSION 2	25.4	26.0	27.7	27.1	27.9	28.7	1.4
A - MT DIFFERENCE	−6.6	−4.2	−1.1	−1.6	0.6	3.2	3.7
L - L’ IMPRESSION 1	27.5	30.5	31.7	31.5	33.3	34.3	2.5
L - L’ IMPRESSION 2	26.3	28.4	28.9	29.2	29.8	33.2	2.3
L - L’ DIFFERENCE	−4.8	−4.5	−4.3	−2.3	−2.1	5.7	4.1
SA - SA’ IMPRESSION 1	5.2	7.8	10.8	11.4	14.3	19.8	5.4
SA - SA’ IMPRESSION 2	2.6	3.2	3.8	5.1	6.9	9.4	2.8
SA - SA’ DIFFERENCE	−10.3	−8.7	−6.0	−6.4	−4.7	−2.0	3.1
T - T’ IMPRESSION 1	32.3	32.9	34.4	34.3	35.6	36.4	1.7
T - T’ IMPRESSION 2	33.8	35.2	35.8	35.8	36.7	37.3	1.3
T - T’ DIFFERENCE	−1.3	−0.8	1.0	1.5	3.7	5.0	2.7
SD - SD’ IMPRESSION 1	11.7	21.4	21.9	21.1	22.1	27.4	5.1
SD - SD’ IMPRESSION 2	16.9	20.0	21.0	20.7	22.5	22.9	2.2
SD - SD’ DIFFERENCE	−4.5	−1.9	−0.5	−0.3	0.5	5.2	3.3

### RapidNAM plate design

**Figure 5:**
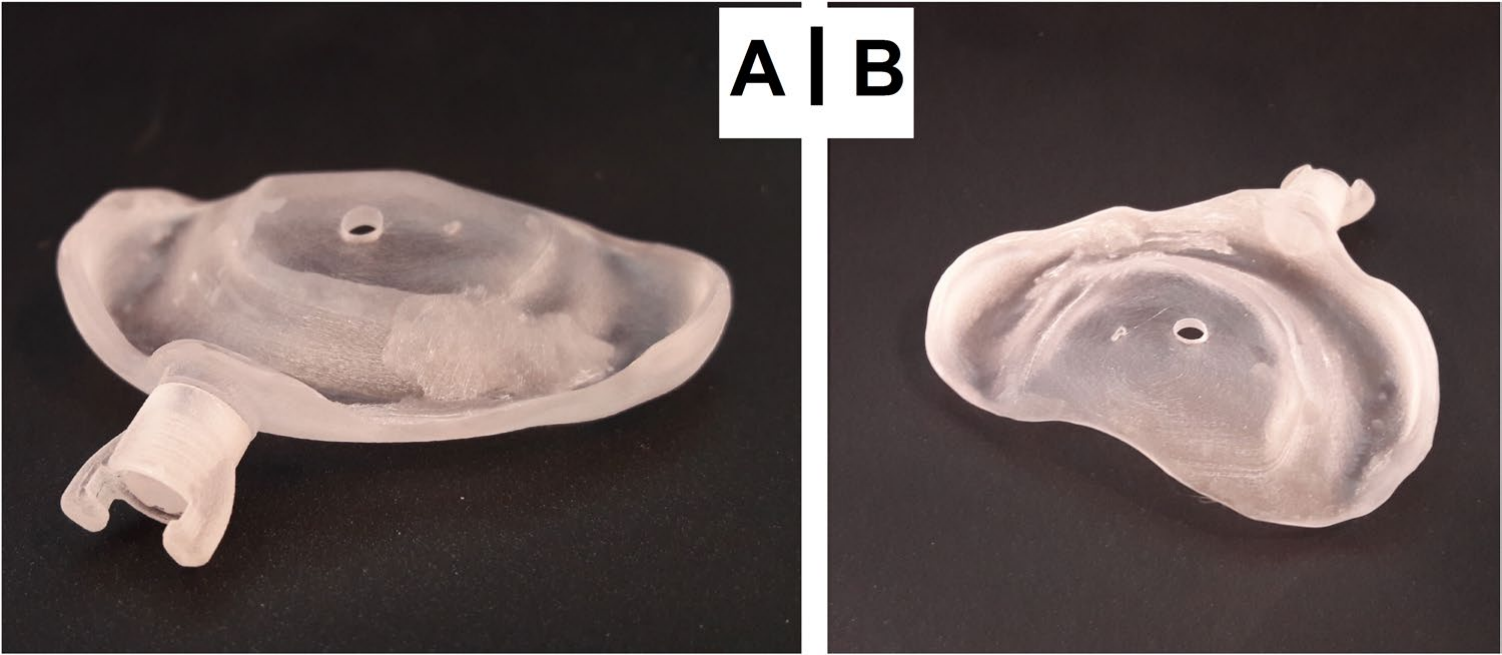
RapidNAM device in frontal (**A**) and dorsal (**B**) view.

Based on only one impression-taking, the RapidNAM-intraoral molding plates had a very good intraoral fit. The change to the next size was handled concordant with conventional intraoral molding, when the preceding plate was losing adhesion. Adhesive cream was recommended for the very first days with the new plate. Systematically, grinding had to be performed at two sights: (1) at the tuberal level bordering the posterior vestibule, where edges had to be smoothed; (2) at the anterior vestibule, where the upper lips are transversally taped and therefore brought closer to the plate around the retention pin. Since the plates are so similar, the plates could be adapted even before insertion. The retention of the buccal taping on the plate was enabled by the retention pin. As for the manual and conventional CAD/CAM-method, an original series of six plates were planned based on the estimated growth to have enough devices in stock. In both techniques, only 3–4 plates were necessary. Figure 5 shows a RapidNAM-plate ready for clinical application.

## Discussion

This study compares two CAD/CAM-assisted techniques for intraoral molding, one that involves conventionally digitally designed plates and a second method that, to our knowledge, is the first published approach of the semi-automated generation of intraoral molding plates (RapidNAM). The measured longitudinal and transversal dimensions showed comparable results in both groups and were also comparable with the clinical results of a previously analyzed cohort treated with traditional intraoral molding^11,22^. Both applied CAD/CAM-techniques significantly closed the cleft between the two alveolar segments, as reflected by the good convergence of the two segments and the decrease in distance between the points L and L’. Slight expansion was observed in the dorsal area of the palatal part of the cleft at the tuberal level. This finding can be explained as the maxilla being given space for expansion without growth restriction. On the assumption of normal growth, close oral inspection was performed weekly, with special attention being paid to any mucosal ulcerations as a possible hint of an unfavorable fit. Pressure marks were easily prevented by grinding the corresponding areas on the plate. The total longitudinal length measured by the distance between A and MT remained almost the same within the observation period in both groups. This can be explained by the initial misalignment of the greater segment, since its anterior orientation points towards the oral orifice. This can be observed by comparing the positon of the A point and the papilla incisiva with a normal maxilla. Aiming at the achievement of a harmonic arch, the plate design guided the growth and expansion of the greater segment as a passive device. The result was a positioning of the A point and the papilla incisiva towards the normal anatomical position in the median line of the maxilla. RapidNAM was oriented on normal postnatal dentoalveolar development and supported by data from a longitudinal growth analysis. However, only 3–4 plates were necessary, despite the calculated 6 plates. This might be explained by, first, the good closure of the cleft resulting in a desired reduction of transversal dimensions, and second, the elliptic alignment of the greater segment aiming for a harmonic arch as present in healthy newborns. The total length of 28.7 mm after RapidNAM was even somewhat greater compared with healthy newborns that reached 24.4 mm after 5 months^19^. This finding may oppose the claimed criticism that molding therapy might restrict maxillary growth^25^. It supports the presumption that maxillary growth deceleration occurs at a later stage after surgical intervention^26^.

Since anatomical developments have been derived from healthy newborns, which represent, in our opinion, the desired symmetry, we have considered our own previously stated objections with regard to digitally designed intraoral molding devices, namely that not only closure, but also the alignment is of importance^27^. Since the virtual closure of the cleft was performed by bridging the gap based on a fitted ellipse, the alveolar growth has a type of guiding track into which it can expand^19^. This kind of free-form molding has previously been implemented in intraoral molding^16^. Throughout therapy, the two alveolar segments did not experience any active rotation attributable to only one impression-taking. This guaranteed that the main orientation of the greater and especially of the minor segment was not actively changed by the intraoral molding device. The usage of CAD/CAM-technology does not mean that the state and the shape of molding devices are unchangeable. Because of the similarity of the materials used in the traditional and the printing means of production, the molding devices combine traditional elements with new technology and therefore stay necessarily very flexible. Adaptations to the plate by removal of material, especially at the cleft lip bordering vermilion, were still necessary and had to be done at the first treatment session. The first plate could then be used as a template, and changes could be transferred to subsequent plates. The clefts sizes treated with RapidNAM were all rather moderate in their extent. The algorithms were able to detect the alveolar ridge in all cases. However, in very broad clefts or difficult clinical mucosal situations, conventionally produced plates might have to be applied with several impression-takings. RapidNAM is therefore not designed as a replacement of traditional nasoalveolar molding, but rather as a simplification for suitable clinical situations.

Use of a semi-automated production of intraoral molding plate does not mean that traditional elements of alveolar molding are excluded. Acrylic resin can be added, or areas on the plate surface to avoid ulcerations can be removed, when necessary. This study is, to our knowledge, the first to report clinical results of a novel semi-automated molding technique as a derivative of feeding-plates, combining conventional elements with rapid-prototyping opportunities. The semi-automated workflow is based on an algorithm that detects the alveolar ridge, and the plate generation is derived from a monthly percentage relative growth factor measured in healthy newborns^19,20^. Compared to conventional molding plates which involved cost up to 223USD per plate, RapidNAM devices cost up to 155USD which excludes a later adjustment by a dental technician when starting nasal molding. This is the case in conventional NAM therapy and would involve extra costs ranging from 66USD to 103USD. Considering only the costs of the presented two CAD/CAM-molding solutions, production costs are similar, however, the semi-automated workflow based on the GUI is far easier to use and has its advantages by considering the time savings during of the plate design. The presented CAD/CAM-intraoral molding techniques both provide the opportunity to reduce repeated impression-takings to a single initial procedure while conventional intraoral molding needs a second or third impression-taking whenever a new plate is needed. In order to show our clinical results we performed further impression-takings at the end of the molding therapy. In regards to future technical developments a reduction of the burden of impression-takings might appear when intraoral scanners will decrease in size and scanning time to become available for quick virtual impression-takings. Since the plate can be designed in several steps that can be adjusted and changed, the presented solution is easy to use and time-efficient^20^. Future tasks include a more detailed plate design at the transition to the vestibule and the upper lips near the retention pin. Further developments for a still easier application and accessibility of the software for other practitioners are planned for a more widespread use of CAD/CAM-molding therapy.

## Conclusions

RapidNAM overcomes previous limitations of conventional CAD/CAM-intraoral molding plates by its semi-automated workflow. The GUI creates a series of molding plates within a few minutes but still allows changes by the user. The resulting plates are as adaptable as conventional NAM-devices. The algorithm automatically detects the edentulous alveolar ridges and may also have further dental applications. RapidNAM gives good clinical results and may bring nasoalveolar molding to a broader practice.

### Ethical Statement and Patient Recruitment

All clinical investigations and procedures were conducted according to the principles expressed in the Declaration of Helsinki. Ethical approval for the prospective application study was granted by the Ethical Committee of the Technische Universität München (Approval No. 67/15S). All interactions with each patient were performed with written parental consent.
