# Author Correction: A semi-automated virtual workflow solution for the design and production of intraoral molding plates using additive manufacturing: the first clinical results of a pilot-study

**DOI:** 10.1038/s41598-020-61778-6

**Published:** 2020-03-10

**Authors:** Florian D. Grill, Lucas M. Ritschl, Franz X. Bauer, Andrea Rau, Dominik Gau, Maximilian Roth, Markus Eblenkamp, Klaus-Dietrich Wolff, Denys J. Loeffelbein

**Affiliations:** 1 Department of Oral and Maxillofacial Surgery, Technische Universität München, München, Germany; 2 Institute of Medical and Polymer Engineering, Technische Universität München, München, Germany; 3 Department of Oral and Maxillofacial Surgery, Friedrich Alexander Universität Erlangen-Nürnberg, Erlangen, Germany; 4 Department of Oral and Maxillofacial Surgery, Helios Hospital Munich West, Teaching Hospital of Ludwig-Maximilians-Universiität München, München, Germany

Correction to: *Scientific Reports* 10.1038/s41598-018-29959-6, published online 07 August 2018

This Article contains errors in Table 2 where the two columns headings have been reversed. ‘CAD/CAM NAM’ and ‘RapidNAM’ should read ‘RapidNAM’ and ‘CAD/CAM NAM’ respectively.

